# Paramagnetic Liposome Nanoparticles for Cellular and Tumour Imaging

**DOI:** 10.3390/ijms11041759

**Published:** 2010-04-15

**Authors:** Nazila Kamaly, Andrew D. Miller

**Affiliations:** Department of Chemistry, Imperial College Genetic Therapies Centre, Imperial College London, Flowers Building, Armstrong Road, London, SW7 2AZ, UK; E-Mail: nazila.kamaly@imperial.ac.uk

**Keywords:** cellular labeling, liposomes, Gadolinium, paramagnetic liposomes, bimodal liposomes, cellular imaging, tumour imaging, MRI, MRI contrast agents, nanoparticles, magnetic nanoparticles

## Abstract

In this review we discuss the development of paramagnetic liposomes incorporating MRI contrast agents and show how these are utilized in cellular imaging *in vitro*. Bi-functional, bi-modal imaging paramagnetic liposome systems are also described. Next we discuss the upgrading of paramagnetic liposomes into bi-modal imaging neutral nanoparticles for *in vivo* imaging applications. We discuss the development of such systems and show how paramagnetic liposomes and imaging nanoparticles could be developed as platforms for future multi-functional, multi-modal imaging theranostic nanodevices tailor-made for the combined imaging of early stage disease pathology and functional drug delivery.

## 1. Introduction

Cellular imaging has been defined as “the visualization of specific cells in an intact animal” but this is also a collective term for the visualisation of any type of whole cell under a variety of circumstances [1]. Hence cellular imaging concerns the imaging of entire cells whereas molecular imaging seeks to visualise molecules and sub-cellular components within individual cells [2]. Both cellular and molecular imaging requires the use of molecular probes (or imaging agents) to facilitate the use and applications of either technique. In the case of molecular imaging, the fluorescence imaging of extrinsic fluorophores is a very useful method, however, the technique is often depth restricted. Additionally, positron emission tomography (PET) or single-photon computed tomography (SPECT) have limitations due to short lived radioisotopes and poor spatial resolution. This is not the case for magnetic resonance imaging (MRI) which is capable of producing three-dimensional images of tissues containing water with a high degree of spatial resolution. However, the main weakness of MRI is an inherent lack of sensitivity which is overcome through the use of contrast agents which enhance signal sensitivity and hence image quality. MRI contrast agents consist of molecules or nanoparticles that incorporate a paramagnetic metal ion, most commonly gadolinium (Gd^3+^) or Iron (Fe^3+^/Fe^2+^). The improvement in image quality that arises with these contrast agents, derives from the modulating effects of the coordinated metal ions on longitudinal (*T*_1_) or transverse (*T*_2_) relaxation times associated with proton resonance signals emanating from bulk water molecules surrounding the coordinated metal ions (see below). Contrast agents incorporating Gd^3+^ increase both 1/*T*_1_ and 1/*T*_2_ relaxivities but are generally used in *T*_1_-weighted (positive-bright) contrast imaging given that the 1/*T*_1_ contribution is greater in tissue than the corresponding effect on 1/*T*_2_ enhancement. On the other hand, Iron containing contrast agents promote more substantial increases in 1/*T*_2_ and are generally used in *T*_2_-weighted (negative-dark) contrast imaging [3]. In the next section we shall look at contrast agents in more detail, focusing on Gd^3+^based systems.

## 2. MRI Contrast Agents

**Figure 1.**
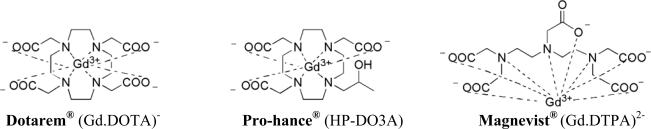
Commonly used Gadolinium based clinical contrast agents approved by the FDA.

MRI contrast agents are routinely used in clinical diagnosis as they provide reliable means to interpret MR images and can also play a key role in clinical imaging [3]. In general, MR contrast agents consist of Gd^3+^ complexes, Mn^2+^ chloride complexes, or monocrystalline iron oxide nanoparticles. In complexes, a paramagnetic metal ion core is associated with a strong chelating ligand. Nowadays, the most frequently used contrast agents are thermodynamically and kinetically stable low molecular weight Gd^3+^ complexes that promote MRI contrast by non-specific enhancement of water proton relaxation rates within the blood pool. Gd.DTPA (Figure 1) was the first water soluble, renally excreteable contrast agent approved for clinical use, and is currently used routinely under the commercial name Magnevist^®^ [4]. Other Gd^3+^ contrast agents used commonly in the clinic are also shown (Figure 1).

These contrast agents are stable complexes with Gd^3+^ ion strongly chelated to poly(aminocarboxylate) ligands. They mostly act non-specifically, mainly reside within the blood stream and also accumulate in the kidneys due to glomerular filtration from where they are excreted unmetabolised [5]. Their use in clinical MRI has been substantial given that anatomical abnormalities, such as gliomas and lesions within the brain, can be visualised with ease. This is due to the fact that the blood brain barrier in these instances is porous to such contrast agents, but not under normal physiological conditions. Pathologies within the liver and other organs can also be visualised too under circumstances where contrast agents are able to accumulate rapidly into regions of increased fluid volume in interstitial spaces thereby leading to increases in local MRI signal to noise ratios and image quality [6].

The Gd^3+^ ion is of particular utility in MRI owing to its high magnetic moment (7 unpaired electrons in its outer shell, 7.9 μ_B_) and slow electron spin relaxation times equivalent to spin relaxation times of structural water protons. The principle mechanism for Gd^3+^ ion-mediated modulation of longitudinal (*T*_1_) or transverse (*T*_2_) relaxation times is due to t the interaction of an inner sphere water molecule with the fluctuating local magnetic field of the paramagnetic Gd^3+^ ion. The inner sphere water molecule coordinates to the 9^th^ coordination site of the Gd^3+^ ion, leading to the subsequent magnetic relaxation of this water molecule. This relaxation has a catalytic effect that leads to the relaxation of surrounding bulk water molecules in the vicinity of this co-ordinated water molecule as well as via a chemical exchange process. The relaxation process can also be envisaged in terms of a Gd^3+^ ion mediated polarisation of the O-H bond of the inner sphere coordinated water molecule that enhances the magnetic moment of this molecule, and hence dynamically affects surrounding bulk water molecules that are in hydrogen bond relationships with the inner sphere water molecule.

In MRI a static magnetic field is applied which polarizes protons in a sample, producing a net magnetisation vector. An RF pulse is then generated which perturbs the net magnetisation vector and changes its direction. The magnetisation vector then precesses at a frequency proportional to the static applied field, producing the NMR signal which is picked up by an RF receiver. By applying gradients to the static magnetic field throughout the sample, the position of each proton atom can be pinpointed through slice selection, phase and frequency encoding to produce an MR image (effectively a proton density map).

After the RF pulse is switched off, the magnetisation vector returns to its original direction dictated by thermal equilibrium. As it does so, the signal decays (Free Induction Decay, FID). By introducing additional random magnetic perturbations Gd^3+^ increases the rate of this thermalization and therefore decreases the FID rate. Signal processing can be used to extract decay rates and construct proton density maps weighted by T_1_ or T_2_. These will effectively translate as concentrations of contrast agents on an MR image.

One way to increase the utility of imaging contrast agents is to ensure the loading of as many Gd moieties in close proximity as possible. This approach has led to the design and synthesis of macromolecular Gd^3+^ containing contrast agents that involve the conjugation of many Gd.DOTA or Gd.DTPA moieties together on linear, branched or dendrimeric macromolecular polymeric structures with powerful capacities to modulate *T*_1_ relaxation [7]. However, their routine use in the clinic may be hampered by slow clearance and inherent toxicity *in vivo*, [8,9] although efforts to make these agents biodegradable and hence less toxic have been undertaken [10,11]. Nevertheless, applications in MR angiography are impressive [12]. Moreover, these macromolecular systems can be bi-functional, that is they act simultaneously as imaging contrast agents and drug delivery systems [13].

## 3. Cellular Labelling and MRI

**Figure 2.**
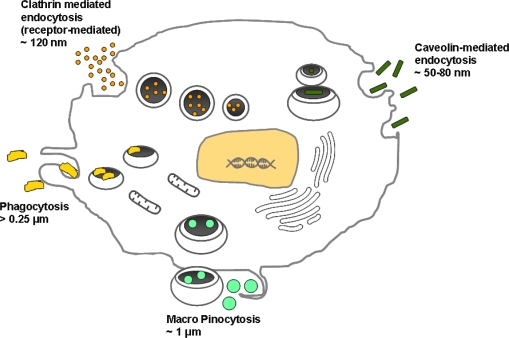
Cell entry mechanisms: the various uptake mechanisms of macromolecules and nanoparticles into cells are indicated according to size.

For cell populations to be imaged by MRI, these populations must be distinguishable from natural background noise signal. The ideal cellular label as addressed by Frangioni *et al.* should posses the following characteristics: have a strong signalling effect, good biocompatibility, not interfere with cellular genetics, remain retained only in the targeted cell population, and finally, enable temporal imaging over long time periods [14]. Clearly cells of interest could be labelled with these agents *in vitro* or *ex-vivo* and then subsequently re-implanted into specific sites *in vivo*. Alternatively, cells of interest could be labelled with appropriate agents *in vivo*. Mechanisms of cell entry are shown (Figure 2) [15].

There are a number of pathways by which macromolecules and nanoparticles could enter mammalian cells, namely by phagocytosis, pinocytosis, clathrin-mediated endocytosis, caveolin-mediated endocytosis, or clathrin and caveolin independent endocytosis [1,16]. Phagocytosis is a process that mediates the uptake of large particles (1 μm) by cells and most phagocytic cells of the immune system use phagocytosis to internalise foreign bodies. These cells include neutrophils, monocytes, macrophages, and microglia. Non-phagocytic cells prefer to internalise particles using pinocytosis and it is generally considered that any non-specific internalisation into cells is due to this mechanism [1]. Clathrin-mediated endocytosis and caveolin-mediated endocytosis are much the more specific means of cellular entry.

Composed of lipids, similar to cellular bilayers, liposomes or lipid-based nanoparticles represent a very useful alternative to macromolecular polymeric structures as carriers of contrast agents, not only because they can be adapted to meet the specific requirements of cell labelling vectors, but also to meet the general criteria for the development of novel potential MRI contrast agents that can be summarized as: *an increased thermodynamic stability*, *favourable rate of excretion*, *lowered toxicity*, *lipophilicity*, *target specific biodistribution and an increase in relaxivity*. Hence the present review will focus on the development of paramagnetic liposomes and imaging nanoparticles with a utility for cellular imaging and cancer imaging *in vitro* and *in vivo*. For completeness sake, we would like to refer the reader to more extensive reviews that concern the syntheses, ligand designs and properties of Gd contrast agents, in particular clinically relevant agents [17,18].

## 4. Liposome Carriers for Imaging Agents

**Figure 3.**
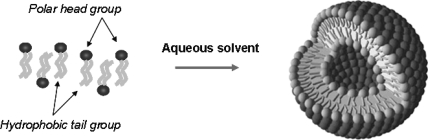
Schematic of liposome self-assembly from lipids.

Liposomes are composed of lipid constituents, with hydrophobic head groups and hydrophilic tail groups (Figure 3). Liposomes are prepared by addition of known molarities of desired lipids together in organic solution, the organic solution is then slowly evaporated *in vacuo* to produce a thin film which is then hydrated with a desired aqueous buffer and sonicated. The sonication technique lifts the film and creates budding vesicles of bilayers enclosing an aqueous cavity, which achieve a stable size once the bilayer has reached a stable equilibrium. The particles are generally on the nanometer scale and can be further size refined by passage through physical membrane pores of known size (extrusion). Extrusion may also be solely used as a method of preparing liposomes, in particular when drug loading capabilities are also required within the liposome aqueous cores.

Liposomes are typically characterised by their size, shape and lamellarity. They may be composed of a single bilayer (unilamellar), a few bilayers (oligolamellar), or multiple bilayers (multilamellar). The rigidity of the membrane can also be modified with the use of suitable lipids; and the fluidity of the membrane may be controlled using phospholipids with higher or lower L_αI_ − H_II_ phase transition temperatures. In general lipid derivatives of steric acids (fully saturated C18 lipidic chains) bestow rigidity and impermeability to the membrane, whilst lipid derivatives of oleic acid (Δ^9^ unsaturated C18 lipidic chains) result in a more permeable and less stable lipid bilayer. Due to their aqueous cavity and “tunable” bilayer, liposomes have traditionally been used as drug delivery vehicles, encapsulating water-soluble drugs within the aqueous cavity in order to improve drug pharmacokinetics [19–21]. Once liposomes are formulated they can be characterized for their size and surface charge. Liposomes can be investigated for their hydrodynamic diameter which is measured using scattered light off the surface of the particles with photon correlation spectroscopy, or using cryo-TEM where a visual image of the shape and size of the liposomes can be obtained. The surface charge of the liposomes can be obtained by measuring their zeta potential (electrokinetic potential).

One particularly important modification of liposomes has been the inclusion of synthetic cationic lipids into bilayers such that cationic liposomes are formed. These cationic liposomes readily combine with nucleic acids to form cationic liposome/nucleic acid (lipoplex) nanoparticles that mediate functional delivery of nucleic acids to cells [22–28]. Otherwise, liposomes *per se* are remarkably biocompatible and have been studied as models of biological membranes [29]. Of particular relevance here, liposomes are rendered MRI active (paramagnetic liposomes) by the incorporation of Gd lipids into the bilayer structures or by the encapsulation of paramagnetic contrast agents within the aqueous cavity [30–32]. These paramagnetic liposomes have been used in a number of investigations including cellular labelling and tracking [33]. Indeed, liposomes appear well suited as carriers of a high payload of Gd^3+^ chelates into cells.

### 4.1. Paramagnetic Liposomes by Contrast Agent Encapsulation

The initial use of paramagnetic MRI-active liposomes came through encapsulation of a Gd^3+^-containing contrast agent in the aqueous core of liposomes (where water-soluble agents were entrapped) or in the bilayer structure (where the lipid soluble agents were concerned) [34–38]. The most common form of paramagnetic liposome systems involves the cavity encapsulation of FDA-approved clinical contrast agent Magnevist (Gd.DTPA) that has excellent solubility and MR signal enhancement properties. Many paramagnetic contrast agents have been prepared in paramagnetic liposomes for a range of applications [39,40], assisted by the work of Unger *et al.* and others who have noted that acceptable encapsulation efficiencies of >35% are obtained using a freezethaw extrusion process [41]. In general relaxivity rates associated with Gd^3+^ ion-mediated *T*_1_-relaxation are a function of the exchange rate (*t*_M_) of the inner and outer sphere water molecules [42]. In addition, the coordination number and the rotational correlation time (*t*_r_) are also factors that affect relaxivity rates [43]. In the case of paramagnetic liposomes, these effects need to be considered alongside other factors such as liposome rigidity that leads to limited water flux between the liposome aqueous cavity and outer bulk water, and can impair the overall impact of the contrast agents on local tissue water relaxivity [44]. Accordingly, lipid properties such as lipid chain length, the presence of a phospholipid head group and degree of saturation [45,46], can be manipulated to engineer paramagnetic liposome associated relaxivity rates. One of the best ways to overcome paramagnetic liposome rigidity can be to prepare very small liposomes (<50 nm), such that a high surface to volume ratio facilitates water exchange. Alternatively, bilayers can be designed to be as permeable as possible to water, but this raises issues of instability in biological media such as serum [40].

### 4.2. Paramagnetic Liposomes Involving Gd Lipid Incorporation

**Figure 4.**
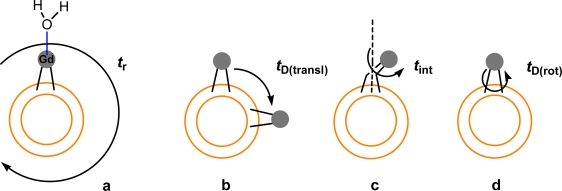
Cartoon of the possible motions that may occur involving the water molecule bound to the Gd^3+^ ion of the Gd lipid chelate head group. a shows the rotational motion of the whole paramagnetic liposome in water (*t*_r_), b shows the translational diffusion of individual Gd chelates protruding from the paramagnetic liposome surface (*t*_D(transl)_), c presents the rotation of the Gd chelated head group attached to the lipid tail (*t*_int_) and d depicts the rotational diffusion of the entire Gd lipid in the paramagnetic liposome membrane (*t*_D(rot)_) [42].

Various parameters exist that can influence the relaxation rate of water molecules within the contrast agent’s vicinity. These parameters can be thought of as; the average time (nanoseconds) a water molecule remains co-ordinated to the metal ion in the first inner sphere (*t*_M_), how fast the contrast agent tumbles in water, which is measured by the rotational correlation time (*t*_R_) and the translational diffusion time of the outer sphere water molecules (*t*_D_). This latter parameter is related to the hydrogen bonding of the carboxylate oxygens with surrounding water molecules that hydrate the complex (oxygen atoms are relaxed via dipolar mechanisms). The relaxivity of paramagnetic liposomes has been shown to be mainly effected by the exchange rate (*t*_M_) of the coordinated water molecule to the Gd metal centre of the membrane anchored paramagnetic lipid. In addition to the exchange rate, the coordination number and the rotational correlation time (*t*_r_) are also factors that affect the relaxivity of macromolecular contrast agents [43]. Possible motions of the enhanced dipole moment of the inner sphere water molecule (associated with the Gd chelate head group) are shown that are considered to contribute towards relaxation of surrounding water protons in Figure 4 [42]. Paramagnetic liposome rigidity (achieved through the use of fully saturated lipids) leads to limited water flux between the inner liposome compartment and outer bulk water, therefore affecting the overall particle relaxivity [44]. Paramagnetic lipid properties such as lipid chain length and degree of saturation [45], in addition to the presence of a phospholipid head group have also been shown to affect relaxivity [46]. Given relaxivity problems resulting from the encapsulation of Gd^3+^ contrast agents inside liposomes, surface attachment of Gd chelates (such as Gd.DTPA or Gd.DOTA) to liposome bilayers now appears increasingly preferable. Surface attachment can be expected to enhance water contact of the Gd chelate leading to potentially significant relaxivity enhancement effects.

### 4.3. Effects of Cellular Compartmentalization of Gd Liposomes on MR Relaxivity

Once paramagnetic liposomes have passed the first hurdle of cellular entry through envagination of the cellular bilayer (endocytosis) and reside in early endosome compartments, the T_1_ relaxation properties of the Gd chelates bound to the lipids becomes limited to the amount of water flux between the endosmal and eventually, lysosomal compartments. Terreno *et al.* have elegantly demonstrated the effects of Gd chelate localization and concentration on relaxation properties, with quenching effects observed when chelates are trapped in the endosome and at high concentrations of Gd^3+^ [47].

When considering the cellular labelling efficiencies of single molecule based contrast agents or liposomes, factors such as the path of entry into the cell, the intracellular localization and concentration of MRI contrast agents must also be taken into account. Lack of sufficient water protons to allow for detectable T_1_ relaxation effects and Gd^3+^ concentrations leading to quenching of the T_1_ signal are parameters which must also be investigated in future assessments of the efficacies of cell labelling contrast agents. Recently a three compartment model representing the extracellular, cytoplasmic and vesicular components of cells has offered a theoretical model for the explanation of these effects once contrast agents are added to cells [48]. Additionally, Kok *et al.* have also demonstrated that intracellular effects described above directly affect T_1_ relaxation properties [49]. In this study RGD targeted liposomes were used to image α_v_β_3_ overexpression on HUVEC cells, in addition to control non-targeted liposomes, and it was shown that despite the additional RGD targeting moiety which should increase liposome uptake into the cells, no changes in T_1_ enhancement were observed compared to when the cells were incubated with control non-targeted liposomes. These findings, which were explained based on the model by Strijkers *et al.* were attributed to; the concentration of Gd^3+^ in the intracellular vesicles post internalization and the physical size of these vesicles [49]. With high contrast agents inside the vesicles limiting water flux between the vesicles and the cytoplasmic compartment, and due to enhanced uptake for the targeted liposomes, an increase in the size of these vesicles leading to an increase in their surface-to-volume ratios, thereby lowering the water exchange of the overall membrane [49].

Kabalka *et al.* were the first to demonstrate how to incorporate Gd lipids into paramagnetic liposome formulations over 20 years ago and the Gd lipid (Gd.DTPA.BSA) used in their studies is still frequently used to prepare paramagnetic liposomes today [50]. Kabalka *et al.* also clearly demonstrated how the attachment of two hydrophobic chains to the DTPA chelate had little effect on the ability of DTPA to complex Gd^3+^ [51]. On the other hand, they also showed that such liposomes containing amphipathic paramagnetic agents (*i.e.*, Gd lipids) were indeed able to enhance significantly, the MR signal intensity in T_1_-weighted MRI. In addition, they were able to observe that their paramagnetic liposomes agents were suitable for the imaging of liver, spleen, bone marrow, and other organs that are rich in macrophage activity.

**Figure 5.**
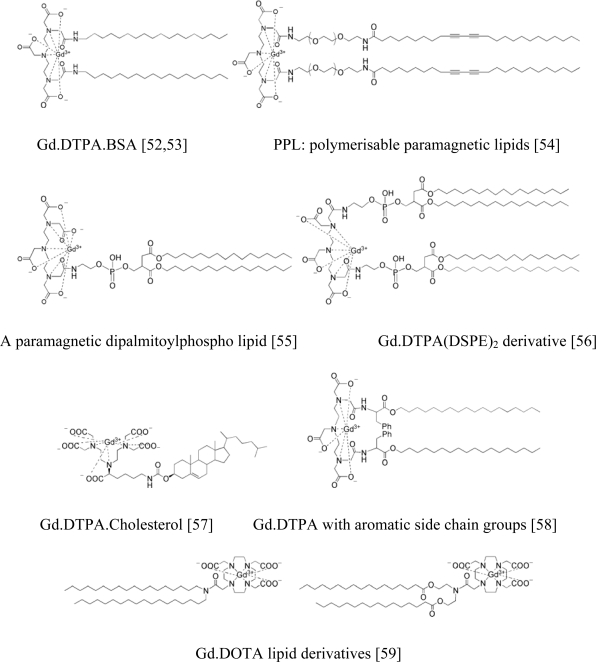
Examples of paramagnetic Gd lipids with various hydrophobic tails.

Since that time, a range of Gd lipids have been incorporated into liposome formulations to achieve paramagnetic liposomes. The principal design feature of these lipids has been the conjugation of the FDA approved Gd.DTPA MRI contrast agent moiety with lipid moieties associated in order to anchor the Gd chelate into liposome bilayers. Some examples of paramagnetic Gd lipids synthesised by various research groups are presented (Figure 5).

**Figure 6.**
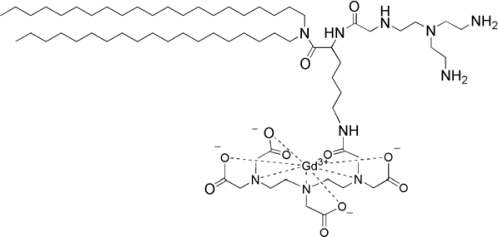
bi-functional Gd lipid MCO-I-68 [60].

Leclercq *et al.* have reported the design and synthesis of an alternative paramagnetic Gd lipid with an additional functionality which further demonstrates additional advantages of liposome systems [60]. This novel bi-functional Gd lipid MCO-I-68 (see Figure 6) comprises a cationic head group for DNA binding and condensation. Consequently, cationic liposome-mediated transfection of NIH 3T3 cells was observed *in vitro* using cationic liposomes formulated from MCO-I-68 and transfection of mouse tumours *in vivo* was also observed following intratumoural injection. In this instance, transfection efficiency was determined throughout using concomitant MRI measurements. This is an excellent early example of a bi-functional paramagnetic cationic liposome system.

## 5. *In Vitro* Evaluation of Paramagnetic Liposomes

**Figure 7.**
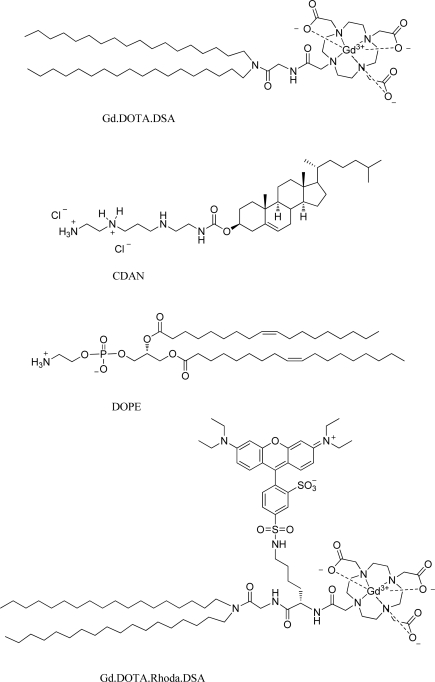
Lipidic agents used to formulate paramagnetic liposome nanoparticles [61].

To date, cellular labelling with MRI contrast agents has frequently involved the use of iron oxide particles. However, Gd^3+^ based contrast agents are increasingly used as well. Contrast agent-mediated cellular imaging is now a growing field for the tracking of cells of interest and even the monitoring of functional cell status as a function of time. As intimated in Section 3 of this review, paramagnetic liposomes represent an excellent means of carriage for the functional delivery of associated MRI contrast agents to cells for labelling purposes. Recently, Oliver *et al.* reported on a bi-functional, bi-modal imaging paramagnetic cationic liposome system (MAGfect™) capable of simultaneous labelling of cells of interest (with fluorescent probe and MRI contrast agent) and functional delivery of DNA to the same cells [33]. This system was further developed by Kamaly *et al.* using a novel paramagnetic lipid termed Gd.DOTA.DSA (Figure 7) in order to derive a new bi-modal imaging paramagnetic liposome system primarily for *in vitro* use [61].

**Figure 8.**
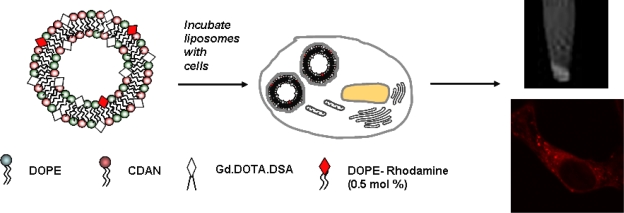
Cellular labelling with bimodal liposomes by MRI (top right image) and fluorescence microscopy (bottom right image).

The cationic character of this particular bi-modal imaging paramagnetic cationic liposome system was conferred by the inclusion of a cationic lipid (CDAN) (Figure 7) that promotes cell surface association, cell entry by endocytosis and endosomolysis to enable liposome bilayer-associated imaging agents access to the cell cytoplasm (see Figure 8). Endosomolysis is further assisted by the lipid fusogenic character of the neutral lipid (DOPE) (Figure 7) that acts as a helper lipid in such membrane trafficking events [62–64]. The inclusion of the cationic lipid was also intended to ensure that this particular bi-modal imaging paramagnetic cationic liposome system should also be bi-functional with the capacity for plasmid DNA binding, condensation and functional delivery to cells *in vitro*, in the same way as MAGfect™ [33,61]. Very recently, Kamaly *et al.* described a next generation bi-modal imaging paramagnetic cationic liposome system prepared using the bi-modal imaging lipid (Gd.DOTA.Rhoda.DSA) (Figure 7) [65]. This bi-modal imaging lipid was found to be even more efficient than the parent Gd-lipid at enhancing water proton relaxivities and lowering *T*_1_ relaxation times.

We have described several paramagnetic cationic liposomes in this Section that are versatile yet robust platforms for the bi-modal labelling of cells of interest (with fluorescent probe and MRI contrast agent) *in vitro*. These same cationic liposomes may also be capable of simultaneous functional delivery of nucleic acids to the same cells too. Accordingly, such systems represent the beginning of new and increasing moves towards the creation of multi-functional, multi-modal imaging paramagnetic liposome systems capable of promoting simultaneous multi-modal imaging of cells *in vitro* and multi-functional adaptations of the same cells. Arguably such multi-functionality/multi-modality should bring to the fore the true technical capacity and value of paramagnetic liposome systems for the cellular imaging of tomorrow.

## 6. Paramagnetic Liposomes for *in Vivo* Applications

For successful *in vivo* applications, experience suggests that an outer liposome bilayer should be coated with a neutral polymer polyethylene glycol; PEG, in order to minimize the colloidal instability of liposomes, to reduce bioadhesion and minimize immunological responses [66,67].

PEG incorporation has also been shown to increase relaxivities of MRI contrast agents through the provision of additional macromolecular bulk structure, leading to reduced tumbling rates and hence increased Gd-metal water contact. In addition, the biocompatibility conferred by a PEG coating has proved valuable in MR imaging of tumours *in vivo* [68], by reducing reticuloendothelial system (RES) mediated uptake and disposal of liposome systems [33], Consequently, PEGylated liposomes may in fact be considered multi-functional nanoparticle systems that can be “engineered” for different applications *in vivo* by variations in lipid composition, liposome size, surface charge, type of constituent lipids, route of injection and injection volume.

Accordingly, PEGylated liposomes that also comprise MRI contrast agents can be expected to be potent imaging nanoparticle systems for *in vivo* MRI applications. This has been demonstrated convincingly in a number of studies. Ayyagari *et al.* have recently reported the encapsulation of contrast agents in PEGylated liposomes [69]. Erdogan *et al.*, and others, have recounted the development of antibody-targeted PEGylated liposomes wherein a polymeric Gd chelate is anchored to liposome bilayers by an attached lipid moiety [70].

Recently Mulder *et al.* have imaged angiogenesis by detecting the over-expression of α_v_β_3_ integrins in tumour bearing mice using a combination of MRI and fluorescence microscopy [71]. They prepared paramagnetic and fluorescent liposomes, surface modified with the cyclic RGD amino acid sequence. These RGD functionalised liposomes were shown to target the vessel walls of cancer cells both *in vitro* and *in vivo.* Fluorescence microscopy revealed that both the control non-paramagnetic and paramagnetic RGD liposomes targeted the vessel wall. The use of fluorescence microscopy in this study differentiated between the activity of the targeted RGD (which strongly bound to the α_v_β_3_ receptor) and the control liposome modified with the control peptide sequence, RAD (Glycine (G) replaced by Alanine (A)), which has no affinity for the receptor.

The versatile nature of liposomes allows for their targeting to various disease markers. Targeting moieties in the forms of ligands, peptides, proteins or specific antibodies can be conjugated onto the surface of liposomes via the use of standard bioconjugation techniques. Gd based paramagnetic liposomes were formulated using the Gd.DTPA.BSA lipid by Brandwijk *et al.* [72]. The incorporation of a maleimide PEG lipid into the liposome formulation allowed for the surface attachment of anginex, a 33-mer angiostatic peptide which has been shown to home to activated endothelium undergoing angiogenesis. MRI and fluorescence microscopy of these targeted liposomes which also incorporated a fluorescent lipidic probe showed specific binding of these agents to activated endothelial HUVECs. A similar approach and liposome model was also used by Mulder *et al.* for the conjugation of an anti-E-selectin monoclonal antibody as a targeting ligand towards activated endothelial cells [73].

In our case, Kamaly *et al.* have described the inclusion of Gd lipid Gd.DOTA.DSA and a Rhodamine-labelled lipid (DOPE-Rhodamine) (Figure 7) into two main types of neutral PEGylated liposomes to give highly effective bi-modal imaging neutral nanoparticles for *in vivo* use. The first such imaging nanoparticle developed (also known as fluorescent Gd^3+^-**BC** nanoparticles according to the **ABCD** nanoparticle concept) [22] was designed for the simultaneous MRI of tumour lesions *in vivo*, fluorescent imaging *ex vivo* and nucleic acid delivery *in vitro* (Gd^3+^-A**BC)** [61]. This system was subsequently adapted for targeting to ovarian xenograft tumours by the PEG surface attachment of folate ligands [74]. The resulting targeted bi-modal imaging neutral nanoparticles (also known as fluorescent Gd^3+^-**BCD** nanoparticles according to the **ABCD** nanoparticle concept) [22] were shown to mediate highly efficient functional delivery of contrast agent and fluorophore to tumour cells within the initial imaging time period of just 2h. Variations of these Gd^3+^-**ABC** and **ABCD** nanoparticles are now the subject of preclinical development and it is hoped that these systems can become genuine clinical-grade imaging nanoparticles for tumour MRI. Recently, a next generation bi-modal imaging neutral nanoparticle (fluorescent Gd^3+^-**ABC** nanoparticle) making use of the bi-modal lipid Gd.DOTA.Rhoda.DSA (Figure 7) to improve further nanoparticle-mediated contrast agent and fluorescence probe labelling of tumour cells *in vivo* was also developed and successfully utilized for *in vivo* tumour imaging [65].

## 7. Conclusions

Here we have set out to summarize recent progress made in the preparation, formulation and investigation of the applications of paramagnetic liposome systems through to imaging nanoparticle systems. These current systems may have real potential to be clinical MRI contrast agents. However this is just the beginning. Once the genuine utility of bi-modal imaging neutral nanoparticles can be demonstrated in the clinic, then we are confident that next steps will be to see the emergence of varieties of multifunctional, multi-modal imaging nanoparticles (nanodevices) ideal for clinical diagnosis of early stage disease pathology, mediation of functional drug delivery (therapy) and even theranostics (where diagnosis and therapy can be realized within one major therapeutic nanodevice). Moreover, the very diversity of lipid structures in nature and available from chemical synthesis should ensure that multi-functional, multi-modal imaging nanoparticles can be truly tailor-made or engineered for individual disease requirements and even individual patient requirements making such devices perfectly applicable to the personalized medicines of tomorrow.
